# Predicting Physical Activity Behavior in African American Females: UsingMulti Theory Model

**Published:** 2018-04-12

**Authors:** Traci Hayes, Vinayak K. Nahar, Manoj Sharma

**Affiliations:** ^1^Behavioral & Environmental Health, School of Public Health, Jackson State University, MS, USA; ^2^Center for Animal and Human Health in Appalachia, College of Veterinary Medicine, DeBusk College of Osteopathic Medicine, and School of Mathematics and Sciences,; ^3^Lincoln Memorial University, Harrogate, TN, USA; ^4^College of Health Sciences, Walden University, Minneapolis, MN, USA

**Keywords:** Exercise, Physical activity, Prevention and control, Methods, Statistical and numerical data

## Abstract

**Background:** Despite physical activity having several benefits, a considerable number of Americans donot engage in sufficient exercise. Among the high-risk groups are African American women. A recenttheory, multi theory model (MTM) for health behavior change can be used to develop effectiveinterventions. The objective of this research was to test MTM in its ability to predict physical activitybehavior in African American women.

**Study design:** A cross-sectional study.

**Methods:** African American women aged 18 yr and older were recruited at various locations (primarilychurches) of Jackson, a large city in Central Mississippi instead of southern Mississippi to participate inthis cross-sectional study in 2016. The valid and reliable survey was administered to a G*Power calculatedquota sample of 156 women either in person or via a Qualtrics link sent through an e-mail.

**Results:** The regression analysis revealed that 32.7% of the variance in initiating physical activity behaviorwas predicted by participatory dialogue, behavioral confidence, and changes in physical environment.Sustenance of physical activity behavior was predicted up to 38.8% by emotional transformation andchanges in social environment.

**Conclusions:** MTM appears to be a robust theory in its application for changing physical activity behaviorin African American women. This theory must be reified and empirically tested with this population.

## Introduction

Adequate physical activity or exercise is associated with many physical and physiological benefits including reducing the risk of several chronic diseases such as diabetes, heart disease, and obesity^1-3^. Individuals with cancer, diabetes, cardiovascular disease, depression, and obesity can benefit from physical activity^3-7^. People who are physically active also tend to live longer ^2^.

According to the 2008 Physical Activity Guidelines for Americans, individuals need two types of physical activity each week to improve and maintain health, namely aerobic and muscle-strengthening^8^. Achieving more than 30 min of vigorous activity for five days during the week along with two days of muscle strengthening exercises is recommended for most adults. Only one in five adults achieves the recommended level of physical activity^8^. Only 18% of African American adults and 16% Hispanic adults meet the requirements compared to 23% of white adults ^2^. Based on CDC’s Health Statistics, 2011-2014, only 36% of African American women are achieving the recommended level of physical activity ^8^.

African Americans are disproportionately impacted by diseases that are reduced through physical activity ^9-11^. They have a higher prevalence of obesity compared with any other ethnic group^10^. They have the highest prevalence of hypertension and diabetes^6^. In general, due to their increased susceptibility to negative health conditions, African American women need to be more physically active^3,5^. Unfortunately, they typically do not meet the recommended level of daily exercise ^3,5,12^.

Researchers have identified social and personal obligations, mental fatigue, unsafe communities, limited fitness facilities, lack of social support and even hair management among the numerous barriers preventing African American females’ physical activity engagement^3,8,12^.

Since African-American are the least likely to volunteer to participate in exercise programs offered in their communities, physical activity interventions should address multiple factors explored at various levels of their environment^9,12^.While theory-based interventions may not work for all, theory-based interventions promoting physical activity behavior change must be adaptive addressing the unique needs of the participant^3,13^. Theory-based interventions were done based on the constructs of social cognitive theory and transtheoretical model to increase the level of physical activity in African American women ^14^. Popular theories are limited when applied to modify health behaviors. Cognitive theories, as related to weight-loss, had not led to better understanding or predictability of weight-loss outcomes ^15^. The best model will account for criterion variance, exhibit determinants of behavior change and has solid predictability ^16^. A criticism of the health belief model and the transtheoretical model was provided, on the basis that they focus only on individual level behavior change ^17^. A new theory was introduced, multi-theory model (MTM) for health behavior change ^17,18^.

The purpose of this cross-sectional study was to assess the utility of MTM to predict physical activity behavior in African American women.

## Methods

### Theoretical Framework

The MTM for health behavior change has been empirically tested with a wide range of behaviors and target populations^19,19-22^. The MTM aims to address both initiation and sustenance of health behavior. MTM has three constructs for initiation of physical activity which are participatory dialogue in which advantages of indulging in a changed behavior outweigh its disadvantages, behavioral confidence, and changes in physical environment. Behavioral confidence is somewhat similar to self-efficacy because the belief and confidence is one’s ability to exercise is partly internal. However, behavioral confidence can also come from external sources^17,18^. Changes to the physical environment entail ensuring the tangible world is prepared and equipped to support the effort to change the behavior. The physical environment must be obtainable, available, accessible, convenient, and provide ready resources ^17,18^.

There are three constructs for sustenance of physical activity or long-term modification of behavior namely emotional transformation, practice for change, and social environment. Emotional transformation relies on the individual’s ability to guide his feelings towards a goal and not succumb to self-doubt. Practice for change consists of the steps or actions such as leaving your gym bag in the car to facilitate going to fitness session. The final construct, change in social environment, entails establishing reinforcement within the immediate supportive environment and building relationships to help goal attainment. The behavior change theory incorporates cognitive, conative, and environmental empirically tested components from existing theories ^17^. The pros of MTM are that it is parsimonious, uses proven constructs, does not have shared variance between constructs, focuses on modifiable constructs, breaks down the behavior change into pragmatic initiation and sustenance (maintenance) components, and is evolving. The cons of this model are that changes in social environment construct are contended by some social psychologists to play a role in initiation model as well, it seems to be a refinement of the popular PRECEDE-PROCEED model, does not have empirical interventional studies to its credit yet and no comparative studies of this model with other models have been undertaken yet ^18^.

### Study Design

This study utilized a cross-sectional design. Since the theory already provided clarity on independent and dependent variables, this design could be used. The independent variables were the constructs of MTM and the dependent variables were the initiation and sustenance of physical activity behaviors.

### Population and Sample

The study was designed to capture a sample of African American women for testing MTM. In this cross-sectional study, African American women aged 18 yr and older were recruited at various locations (primarily churches) of Jackson, a large city in Central Mississippi during a six-month period in 2016. The adult participants did not have any medical conditions to prevent them from being physically active and had not engaged in 150 min or more of physical activity in the past week. There were screening questions in this regard. The valid and reliable survey was administered to a quota sample of 156 women either in person or via a Qualtrics link sent through an e-mail. Sample size was estimated using G*Power as 141 with an alpha of 0.05, power of 0.80, effect size of 0.08 (medium) and number of predictors as the three constructs for each of the two models.

All participants completed an informed consent and the study received approval from the Jackson State University Institutional Review Board.

### Instrument

This study utilized a 38-item valid and reliable questionnaire. The survey instrument was developed for college students and adapted for African American women ^20^. Face and content validity of the questionnaire had been established by a panel of experts (n=6) in the area of health behavior, health promotion, and health education. Flesch-Kincaid grade level of the study instrument was 6.3. Demographics assessed were age, education, income, and working status. The constructs of the multi-theory model for health behavior change was assessed by the following questions.

Advantages component of participatory dialogue were assessed with the five questions. The participants were asked if you participate in more than 150 min of moderate to vigorous intensity aerobic physical activity every week you will “be healthy”, “be relaxed”, “get sick less often”, “have more energy”, and “enjoy life more”. The choices ranged from never (=0) to always (=4). The scores for each question were summed to obtain a total possible score for advantages (ranging from 0 to 20).

Disadvantages component of participatory dialogue were assessed with the five questions. The participants were asked if you participate in more than 150 min of moderate to vigorous intensity aerobic physical activity every week you will “be tired”, “not have enough time for work”, “not have enough time for leisure”, “have to pay for facilities”, and “get injuries”. The choices ranged from never (=0) to always (=4). The scores for each question were summed to obtain a total possible score for disadvantages (ranging from 0 to 20). The difference of the advantages minus disadvantages established the score for construct one, participatory dialogue.

The second construct, behavioral confidence was assessed with five questions. For example, “How sure are you that you will be aerobically physically active with moderate to vigorous intensity for 150 min this week?” The choices ranged from not sure at all (=0) to completely sure (=4). The scores for each question were summed to obtain a total possible score for behavioral confidence (ranging from 0 to 20).

The environment for which the participants were likely to exercise was assessed with three questions. Participants provided input on the likelihood of accessing an exercise facility, utilizing the equipment in a fitness facility and being able to afford a desirable work out location. The responses ranged from not at all sure (=0) to completely sure (=4). The scores for each question were summed to obtain a total possible score for physical environment (ranging from 0 to 12).

In order to assess initiation construct, participants were asked: “How likely is it that you will increase your aerobic PA to 150 min in the upcoming weeks?” Response to this item ranged from not at all likely (=0) to completely likely (=4).

Emotional transformation was assessed using three questions. For the construct of emotional transformation, the participants were asked: “How sure are you that you can direct your emotions/feelings to the goal of being aerobically physically active for 150 min every week?” Participants selected not sure at all (=0) to completely sure (=4). The scores for each question were summed to obtain a total possible score for emotional transformation (ranging from 0 to 12).

Practice for change was assessed using three questions. The practice for change construct was determined by asking the participants how likely they felt about regulating their own self-doubt or motivating themselves to overcome barriers to physical activity. Participants selected not sure at all (=0) to completely sure (=4). The scores for each question were summed to obtain a total possible score for practice for change (ranging from 0 to 12).

To access changes in social environment construct, three questions required the participants to answer how sure they were about acquiring help towards sustaining the physical activity. Participants selected not sure at all (=0) to completely sure (=4). The scores for each question were summed to obtain a total possible score for social environment (ranging from 0 to 12).

The final question, “How likely is it that you will increase your aerobic PA to 150 min every week from now on?” assessed sustenance or long-term implementation of aerobic physical activity. Response to this item ranged from not at all likely (=0) to completely likely (=4).

The face and content validity were established by a six-member panel (n=6). Upon their review, the instrument was revised slightly. Confirmatory factor analysis (CFA) was applied to test construct validity^20^. The Cronbach’s alphas were calculated for each subscale to determine the reliability.

### Data Analyses

The independent variables were the constructs of MTM operationalized as interval/ratio scores and dependent variables were initiation and sustenance of physical activity behavior also operationalized as interval/ratio score. Stepwise multiple regressions were utilized to analyze the survey data. Descriptive statistics were used to analyze the demographic information and describe the data using frequencies and percentages for categorical variables and means and standard deviations for metric variables. All data were analyzed using IBM SPSS (ver. 23 (Chicago, IL, USA).

## Results

The descriptive statistics are summarized in Table 1. A total of 156 participants submitted the completed survey. The participants were all African-American women ranging, 18 yr of age to 80 years. The average age was 42.67 yr (SD: 11.21). Moreover, 92.3% (n=144) of the women were employed. The participant’s education level varied from some high school (n=3; 1.9%) to graduate/professional training (n=69, 44.2%). Almost 38% indicated income less than $40000 per annum.

**Table 1:** Socio-demographic characteristics of the participants, n=156

**Socio-demographics**	**Number**	**Percent**
**Working Status**		
Employed	144	92.3
Unemployed	10	6.4
**Education**		
Some high school	3	1.9
High school graduate	5	3.2
Some college	29	18.6
College graduate	50	32.1
Graduate/Professional	69	44.2
**Income ($)**		
<40000	57	37.5
40000-64999	54	35.5
65000-79999	12	7.9
80000-100000	16	10.5
>100000	13	8.6

Regarding construct validity, all subsets produced one-factor solution with an Eigenvalue greater than 1 and substantive factor loadings, except disadvantages of physical activity. However, disadvantages scale has been found construct valid in previous study, so no changes were made. Cronbach’s alpha coefficients ranged from 0.57 to 0.93 (Table 2).

**Table 2:** Cronbach’s alpha coefficients, n=156

**Constructs**	**Cronbach’s Alpha**
Participatory Dialogue: Advantages	0.85
Participatory Dialogue: Disadvantages	0.57
Behavioral Confidence	0.86
Change in Physical Environment	0.80
All constructs of Initiation Model	0.78
Emotional Transformation	0.93
Practice for Change	0.83
Change in Social Environment	0.74
All Constructs of Sustenance Model	0.90
All Constructs	0.90

Descriptive statistics for constructs of the MTM are presented in Table 3. The mean of 13.55 units (SD: 3.76) for the construct of advantages suggests that participants’ attitude towards physical activity was moderately beneficial and would be likely to initiate behavior change to incorporate physical activity. For the construct of disadvantage, the mean of 7.73 units (SD: 3.16) suggests the participants recognized relatively fewer disadvantages for being physically active. Based on a mean of 8.54 units (SD: 4.93) denotes that participants were somewhat confident in their ability to engage in physical activity. A mean score of 6.72 units (SD: 3.56) for the changes in physical environment construct denotes the participants’ unlikelihood to change the environment to work out. The initiation of behavior means score was 1.75 units (SD: 1.79, range 0-4) which demonstrate that the participants were somewhat likely to start engaging in physical activity in the upcoming weeks. Directing their emotions to engage in physical activity was demonstrated by a mean score of 6.40 units (SD: 3.36) for emotional transformation. The construct of practice for change means score was low at 4.88 units (SD: 3.13) suggesting that the participants were not sure if they were prepared to maintain physical activity. A mean score of 4.72 units (SD: 3.20) for the construct of changes in social environment demonstrated a moderate level of comfort obtaining help and support from friends and family members to become involved in physical activity. The sustenance behavior had a mean of 1.65 units (SD: 1.15, range 0-4) which denote that the participants would be less likely to maintain the required 150 min of aerobic physical activity on a long-term basis.

**Table 3:** Descriptive statistics for constructs of the multi-theory model (MTM), n=156

**Constructs**	**Possible range**	**Observed range**	**Mean**	**SD**
Initiation of Physical Activity	0 to 4	0 to 4	1.75	1.79
Participatory Dialogue: Advantages	0 to 20	0 to 20	13.55	3.76
Participatory Dialogue: Disadvantages	0 to 20	0 to 16	7.73	3.16
Participatory dialogue: advantages - disadvantages score	-20 to 20	-12 to 17	5.81	4.87
Behavioral Confidence	0 to 20	0 to 20	8.54	4.93
Change in Physical Environment	0 to 12	0 to12	6.72	3.56
Sustenance of Physical Activity	0 to 4	0 to 4	1.65	1.15
Emotional Transformation	0 to 12	0 to 12	6.40	3.36
Practice for Change	0 to 12	0 to 12	4.88	4.88
Change in Social Environment	0 to 12	0 to 12	4.72	4.72

The results of the regression analysis of the initiation model and sustenance model are summarized in Table 4. All MTM construct variables were significant for initiation of behavior. Approximately 32.7% of the variance in physical activity initiation was identified through the multiple regression analysis. The three constructs of advantages outweighing the disadvantages or participatory dialogue, behavioral confidence and changes in physical environment were appropriate for explaining the initiation of physical activity among the African-American participants. Emotional transformation and changes in social environment as items explained 38.8% of the variance in physical activity sustenance.

**Table 4:** Parameter estimates for the final regression model for initiation and sustenance of physical activity as predicted by constructs of Multi-Theory Model (MTM), n= 156

**Variables**	**B**	**SE**_B_	**β**	***P*** **-value**
Initiation of physical activity				
Advantages minus Disadvantages	0.038	0.017	0.156	0.029
Behavioral Confidence	0.100	0.017	0.410	0.001
Changes in Physical Environment	0.072	0.024	0.216	0.004
Sustenance of physical activity				
Emotional Transformation	0.166	0.024	0.492	0.001
Practice for Change	--	--	--	0.109
Changes in Social Environment	0.082	0.025	0.230	0.001

R square (Adjusted R^2^) for initiation and sustenance of physical activity was 0.340 (0.327) and 0.396 (0.388), respectively B= unstandardized coefficient; SEB = standard error of the coefficient; β = standard coefficient

## Discussion

The purpose of this study was to test to what extent MTM could predict physical activity behavior in African American women. The MTM successfully predict physical activity among the sample of 156 African American women. The findings of the empirical testing showed that all constructs for physical activity initiation were predictive for initiation of physical activity behavior, which is in consonance with a previous study ^20^. All the three constructs of MTM were successful in predicting substantial amount of variance (32.7%) in intent for initiating physical activity in sedentary African American women. The physical activity sustenance model resulted in only two of the constructs: emotional transformation and changes in social support. The construct of practice for change was not predictive for sustaining physical activity. The two constructs from sustenance model were significant and accounted for substantial proportion of variance in intent to continue physical activity behavior. The construct of practice for change was not significant due to measurement error or maybe this construct is not that important for this target population. African American women in this study were aware of the advantages and disadvantages of physical activity and therefore the construct was an acceptable predictor of PA initiation. Addressing other factors such as time management and access to facilities were essential to successful PA interventions and programs ^9^. An association was found between neighborhood characteristics and the level of physical activity among its African American participants ^23^. The research found a direct correlation between increasing neighborhood median income with increasing physical activity and indirect correlation between increases in the number of renters in the neighborhood with low physical activity among the participants ^9^. Existing research reinforces the current study finding of the MTM construct, changes in physical environment, being significant for the initiation of physical activity. The physical environment for conducting exercise affects whether African American women are obliged to initiate PA.

The construct of behavioral confidence is similar to self-efficacy. Self-efficacy was frequently related to physical activity and that future intervention focused on increasing physical activity should target self-efficacy ^24^. Self-efficacy was a critical component of changing behavior, particularly with physical activity ^25-27^. One’s confidence for implementing PA is important and that was evident in the current study. Once the individual has started engaging in physical activity, it is necessary to promote a path for continuing to be active. Emotional transformation refers to directing one’s feeling to remain committed and focused on the goal ^17^. Emotional transformation showed as a predictor of sustained physical activity among the African American participants. While studying the relationship between loneliness and physical activity, an association between loneliness and one’s ability to regulate and control emotions was identified ^28^. An individual with low ability to regulate one’s emotions was associated with loneliness but also a low level of physical activity. Further evidence was given to the need for social support and engagement for African American women seeking to participate and maintain physical activity ^28^. Several studies document the role of social support for sustaining physical activity among women ^29-32^. In the current study, the construct of changes in social support was significant for predicting physical activity maintenance among African American women. African American women are more likely to engage in exercise if they have support from family and friends or simply being free of adverse and negative criticism from their social circles ^29,32^.

The three constructs of initiation and two constructs of the sustenance models accounted for a significant percentage of the variance in physical activity behavior among the African American female participants. The multi-theory model is a useful framework for predicting physical activity health behavior change and may be suitable for other target populations as also shown in a sample of college students ^20^.

This study also has some potential deficiencies acknowledged. It focused on African American women, which may not exactly reflect response of women in other ethnic groups. More such studies should be carried out across the US with women of other ethnicities. The non-representative sample limits the generalizations made. Moreover, because of self-reported nature of data, influence of social desirability bias cannot be ruled out. Additionally, as a self-report study, some participants included false or exaggerated responses. African American women have varying attitudes towards physical activity compared to others and often will not see the value of exercise as a mandatory part of their day. Finally, due to cross-sectional design of this study, one cannot draw causality or directionality between MTM variables and physical activity among African American women. The participants-initiated exercises and factors to maintain the physical activity are circumstantial. In future, longitudinal designs should be considered to provide more concrete evidence of cause and effect relationships.

## Conclusions

Studies exploring barriers to and motivators for physical activity participation in African Americans have found a variety of personal, interpersonal, and environmental factors influence physical activity behavior in this population. The MTM for health behavior change is effective at the individual, group, and community levels ^17^. Researchers uniting the best components or constructs of the various models and integrating them is an approach to theory evolution ^16^. The MTM successfully explained nearly 40% of the variance between the independent variables and dependent variable. It is not enough to examine the likelihood of behavior change by simply looking at individual choices but including in the theory, other factors such as the environment and systems in which the individual functions ^33^. African American women face specific barriers that prevent their involvement in exercise^9^. MTM appears to be a robust theory suitable for serving as a framework for addressing the various factors impacting health decisions for the population. The MTM for health behavior change may be acceptable for designing physical activity programs for African American women. Behavior change interventions based on the MTM can build upon constructs that affect the individual’s confidence level, the social connections, and physical environment. The constructs of MTM are helpful in both initiation and sustenance of physical activity behavior in African American women and must be verified in future interventions. Future studies need to look at these constructs applied to community-based, culturally appropriate interventions for African American women as well as other subsets of the population.

## Acknowledgements

Authors wish to express their gratitude to the participants, churches, pastors of the churches, and School of Public Health at the Jackson State University for helping with this study.

## Conflict of interest statement

None of the authors has a conflict to disclose as it relates to this educational activity. However, Dr. Manoj Sharma is mentioned as the originator of the Multi-Theory Model being evaluated in the research.

## Funding

No commercial or sponsor support was used for this educational activity.

## Highlights

- African American women typically do not meet the recommended level of daily exercise.
- MTM may be acceptable for designing physical activity programs for African American women.
- Participatory dialogue, behavioral confidence, and changes in physical environment predicted initiation of physical activity.
- Emotional transformation and changes in social environment predicted sustenance of physical activity.

## References

[1] Allen K, Morey MC. Physical activity and adherence. In: Bosworth, H, editor. Improving Patient Treatment Adherence. New York: Springer; 2010. pp. 9-38.

[2] Centers for Disease Control and Prevention, National Center for Chronic Disease Prevention and Health Promotion, Division of Population Health. BRFSS Prevalence & Trends Data. CDC Web Site, 2015 [updated 13 Sep 2017; cited Sep 17]. http://www.cdc.gov/brfss/brfssprevalence/.

[3] Schoeny M E, Fogg L, Buchholz SW, Miller A, Wilbur JE (2017). Barriers to physical activity as moderators of intervention effects. Prev Med Rep.

[4] Dash C, Makambi K, Wallington SF, Sheppard V, Taylor TR, Hicks JS (2015). An exercise trial targeting African-American women with metabolic syndrome and at high risk for breast cancer: Rationale, design, and methods. Contemp Clin Trials.

[5] Sheppard VB, Hicks J, Makambi K, Hurtado-de-Mendoza A, Demark-Wahnefried A, Adams-Campbell L (2016). The feasibility and acceptability of a diet and exercise trial in overweight and obese black breast cancer survivors: The Stepping STONE study. Contemp Clin Trials.

[6] Smalley KB, Warren JC, McClendon S, Peacock W, Caro M (2016). Ethnic identity attachment and motivation for weight loss and exercise among rural, overweight, African-American women. Clin Med Insights Womens Health.

[7] Soltanian AR, Nabipour I, Akhondzadeh S, Moeini B, Bahreini F, Barati M (2011). Association between physical activity and mental health among high-school adolescents in Boushehr province: A population-based study. Iran J Psychiatry.

[8] U.S. Department of Health and Human Services. Physical Activity Guidelines Advisory Committee Report, 2008. [updated 16 Jan 2017; cited 16 Jan 2017] Available from: www.health.gov/paguidelines.

[9] Joseph RP, Ainsworth BE, Keller C, Dodgson JE (2015). Barriers to physical activity among African American women: An integrative review of literature. Women Health.

[10] Rosenberg L, Kipping-Ruane K, Boggs DA, Palmer JR (2013). Physical activity and the incidence of obesity in young African American women. Am J Prev Med.

[11] Sebastião E, Chodzko-Zajko W, Schwingel A (2015). The need to modify physical activity messages to better speak to older African American women: A pilot study. BMC Public Health.

[12] Gothe NP, Kendall BJ (2016). Barriers, motivations, and preferences for physical activity among female African American older adults. Gerontol Geriatr Med.

[13] Rostami Moez, M M, Hazavehei Hazavehei, SMM SMM, Moeini B, Roshanaei G, Taheri M (2014). Applying BASNEF model for predicting regular physical activity of female high school students in Hamadan: A cross- sectional theory based study. Journal of Zanjan University of Medical Sciences and Health Services.

[14] Pekmezi D, Marcus B, Meneses K, Baskin ML, Ard JD, Martin M (2013). Developing an intervention to address physical activity barriers for African-American women in the deep south (USA). Women Health.

[15] Jeffery RW (2004). How can Health Behavior Theory be made more useful for intervention research?. Int J Behav Nutr Phys Act.

[16] Schwarzer R (2008). Modeling health behavior change: How to predict and modify the adoption and maintenance of health behaviors. J Appl Psychol.

[17] Sharma M. Multi-theory model (MTM) for health behavior change. WebMD Central Behaviour Web Site; 2015 [updated 23 September 2015; cited 10 Aug 2016]. Available from: http://www.webmedcentral.com/article_view/4982.

[18] Sharma M. Theoretical foundations of health education and health promotion. Burlington, MA: Jones and Bartlett Learning; 2017.

[19] Knowlden AP, Sharma M, Nahar VK (2017). Using multi-theory model of health behavior change to predict adequate sleep behavior. Fam Community Health.

[20] Nahar K, Sharma M, Catalano HP, Ickes MJ, Johnson P, Ford A (2016). Testing multi-theory model (MTM) in predicting initiation and sustenance of physical activity behavior among college students. Health Promot Perspect.

[21] Sharma M, Catalano HP, Nahar VK, Lingam L, Johnson P, Ford MA (2016). Using multi-theory model to predict initiation and sustenance of small portion size consumption among college students. Health Promot Perspect.

[22] Sharma M, Petosa RL. Measurement and Evaluation for Health Educators. Burlington, MA: Jones & Bartlett Learning, LLC; 2014.

[23] Jones A, Paxton RJ (2015). Neighborhood disadvantage, physical activity barriers, and physical activity among African American breast cancer survivors. Prev Med Rep.

[24] Dishman RK, Motl RW, Saunders R, Felton G, Ward DS, Dowda M (2004). Self-efficacy partially mediates the effect of a school-based physical-activity intervention among adolescent girls. Prev Med.

[25] Lee LL, Arthur A, Avis M (2008). Using self-efficacy theory to develop interventions that help older people overcome psychological barriers to physical activity: A discussion paper. Int J Nurs Stud.

[26] McAuley E, Blissmer B (2000). Self-efficacy determinants and consequences of physical activity. Exerc Sports Sci Rev.

[27] Williams SL, French DP (2011). What are the most effective intervention techniques for changing physical activity self-efficacy and physical activity behavior- and are they the same?. Health Edu Res.

[28] Hawkley LC, Thisted RA, Cacioppo JT (2009). Loneliness Predicts Reduced Physical Activity: Cross-Sectional & Longitudinal Analyses. Health Psychol.

[29] Jackson H, Yates BC, Blanchard S, Zimmerman LM, Hudson D, Pozehl B (2016). Behavior-Specific Influences for Physical Activity Among African American Women. West J Nurs Res.

[30] Mama S, Diamond PM, McCurdy SA, Evans AE, McNeill LH, Lee RE (2015). Individual, social and environmental correlates of physical activity in overweight and obese African American and Hispanic women: A structural equation model analysis. Prev Med Rep.

[31] Miles IW, Kruger J, Liao Y, Carlson SA, Fulton JE (2011). Walking increases among African American adults following a community-based physical activity intervention: Racial and ethnic approaches to community health, 2002–2005. J Health Dispar Res Pract.

[32] Mama S, McNeill LH, McCurdy SA, Evans AE, Diamond PM, Adamus-Leach HJ (2015). Psychosocial Factors and Theory in Physical Activity Studies in Minorities. J Health Dispar Res Pract.

[33] Blue S, Shoveb E, Carmonac C, Kelly MP (2016). Theories of practice and public health: understanding unhealthy practices. Crit Public Health.

